# Prevalence of free flap failure in mandibular osteoradionecrosis reconstruction: a systematic review and meta-analysis

**DOI:** 10.1038/s41598-024-61862-1

**Published:** 2024-05-15

**Authors:** Evangelos Kostares, Michael Kostares, Georgia Kostare, Athanasios Tsakris, Maria Kantzanou

**Affiliations:** 1 Department of Microbiology, Medical School, National and Kapodistrian University of Athens, 115 27 Athens, Greece; 2 Department of Anatomy, Medical School, National and Kapodistrian University of Athens, 115 27 Athens, Greece

**Keywords:** Epidemiology, Oral diseases

## Abstract

Our study aimed to estimate the prevalence of total free flap failure following free flap reconstruction for mandibular osteoradionecrosis (mORN) and assess the impact of potential moderators on this outcome. A comprehensive systematic literature search was independently conducted by two reviewers using the Medline, Scopus, Web of Science and Cochrane Library databases. Quality assessment of the selected studies was performed, and prevalence estimates with 95% confidence intervals (CI) were calculated. Outlier and influential analyses were conducted, and meta-regression analyses was employed to investigate the effects of continuous variables on the estimated prevalence. Ultimately, forty-six eligible studies (involving 1292 participants and 1344 free flaps) were included in our meta-analysis. The findings of our study revealed a prevalence of 3.1% (95% CI 1.3–5.4%) for total free flap failure after reconstruction for mORN. No study was identified as critically influential, and meta-regression analysis did not pinpoint any potential sources of heterogeneity. These findings provide valuable insights for researchers and serve as a foundation for future investigations into the management of mandibular osteoradionecrosis and the prevention of free flap failure in this context.

## Introduction

Head and neck cancer constitutes a heterogeneous group of malignancies, derived from the lips and oral cavity, the salivary glands, the nasopharynx, the oropharynx, the hypopharynx and the larynx with an estimation of 377.713, 53.583, 133.354, 98.412, 84.254 and 184.615 new cases in 2020, respectively^1,2^. Early or advanced stages of oral and oropharyngeal cancers could be benefited from definite or adjunctive ionizing radiation therapy^3^. Under not specified timeframe, mandibular osteoradionecrosis (mORN) may occur, consisting of a serious complication which can adversely affect the patients’ quality of life, functionality and both personal and healthcare expenditure^4,5^. Osteoradionecrosis (ORN) of the jaws is characterized by the presence of exposed bone that has been subjected to radiation and does not show signs of healing for at least three months, in the absence of any ongoing or recurring cancer. When ORN is diagnosed, the bone damage may be either superficial or extensive, and the condition can either progress gradually or rapidly advance, potentially resulting in a pathological fracture^6^. The prevalence of ORN varies considerably in the scientific literature, ranging from 0.4 to 56% and it can manifest spontaneously or induced by a mechanical injury (e.g. tooth extraction)^6^. Regarding the pathophysiological mechanism involved, Marx suggests that the radiation exposure could lead to mandible’s microvascular thrombosis, endothelial death and surrounding tissue fibrosis, causing tissue breakdown and eventually non healing wound formation^7^. Many risk factors that could contribute to its demonstration have been identified throughout the years such as high dose of ionizing irradiation, pre radiotherapy surgical treatment, primary tumor site, trauma (dental extraction), smoking status, male sex, older age, alcohol consumption and poor dental hygiene^5,8,9^ Among the great variety of signs and symptoms of mORN that may occur, an unhealed painful oral lesion is the predominant one. Others include dysaesthesia, odynophagia, malocclusion, trismus, pathological fractures, infections and orocutaneous fistula formation^6^. The main diagnostic procedure used, is a combination of patient’s medical history and clinical examination, therefore the physician should be able to identify all the relevant clinical findings. Radiographic imaging (panographic radiographs, computed tomography of the mandible) as well as biopsy of the wound, could only be used as methods in establishing the diagnosis. Regarding the treatment of mORN, conservative treatment, surgical intervention (minor or major) and hyperbaric oxygen (HBO) are the available options^5^. Resection of the affected tissue and microvascular free flap reconstruction comprise the standard of care for severely affected cases of mORN. Mainly, the free tissue could be harvested through the fibula, iliac crest, scapula and the forearm. However, as with any surgical intervention many complications could arise such as total free flap failure, partial free flap failure and recurrence of mORN. To gain an accurate and reliable estimation regarding the prevalence of the free flap failure after free flap reconstruction for mORN, we conduct a systematic review and meta-analysis using previous data from published studies.

## Materials and methods

### Search strategy

The Medline (PubMed search engine), Scopus, Web of Science and Cochrane Library database were comprehensively searched following the Preferred Reporting Items for Systematic Reviews and Meta-Analysis (PRISMA) guidelines to ensure a rigorous approach^10^. The PRISMA checklist, available in Supplementary materials (Supplementary Table 1), was utilized to facilitate the systematic review process. We have collected articles that were published up until February 27th, 2024. Two reviewers independently conducted the literature search, employing a combination of the following keywords: “*osteoradionecrosis*”, “*mandible*”, “*mandibular*”, “*free flap*”, “*microvascular flap*”, “*free tissue*” and “*reconstruction*” The complete search algorithm for each database can be found in the supplementary materials (Supplementary Table 2). In conjunction with the primary search, a thorough examination of the reference lists from the identified studies was conducted to identify any additional articles that may have been overlooked. The collected studies were meticulously organized and stored using the Zotero reference management software (version 6.0.18)^11^. We ensured the credibility of our dataset by diligently removing any duplicate references. Following the initial search, two independent investigators thoroughly examined the remaining articles. The study selection process consisted of two distinct stages. Initially, we meticulously reviewed the titles and abstracts of the articles, eliminating those that did not meet our predetermined criteria for inclusion. In the second stage, we obtained the full texts of the remaining articles and conducted a comprehensive evaluation. Any disagreements during the study selection were resolved through consensus among the team members, ensuring a consistent and unified decision-making process. By employing this systematic approach, our goal was to obtain a comprehensive and dependable collection of studies for our analysis.

### Criteria for study selection and data extraction

Following our systematic and comprehensive search across multiple databases, we meticulously defined our eligibility criteria using the PECOS framework to ensure clarity and precision in our systematic review and meta-analysis focusing on the prevalence of free flap failure in mandibular osteoradionecrosis reconstruction. Our review includes:

*Population (P)* Adult patients suffering from mandibular osteoradionecrosis who underwent reconstruction using free flap techniques. This population was chosen to assess the efficacy of free flap reconstruction in patients affected by osteoradionecrosis.

*Exposure (E)* The exposure of interest for our study was the utilization of free flap surgical techniques for the reconstruction of the mandible in patients with osteoradionecrosis. This included a variety of flap types, with a specific focus on the prevalence of flap failure post-surgery.

*Comparison (C)* Given that our objective was to quantify the prevalence of free flap failure, a direct comparison component does not apply to our study's framework.

*Outcomes (O)* The main outcome of interest was the prevalence of free flap failure, which was assessed through complete flap losses as reported in the literature. Additionally, we sought to identify risk factors contributing to flap failure to inform better clinical decisions.

*Study Types (S)* Our inclusion criteria encompassed solely observational studies, including cohort, case–control, and cross-sectional studies.

*Exclusion criteria* We opted to omit certain categories of articles from consideration. These exclusions comprised case reports, case series involving fewer than five participants, review articles, randomized and non-randomized clinical trials, systematic reviews, meta-analyses, animal studies, letters to the editor, books, expert opinions, conference abstracts, studies not written in English, articles lacking an adequate follow-up period, studies involving reconstruction using pedicled flaps, studies involving patients with medication-related osteonecrosis, studies that did not specify the patients from whom the free flaps failed^75,76^, studies using populations with multiple pathologies requiring free flap reconstruction without specifying who received a second free flap^77^, and articles that did not clearly mention the failure^78^, studies lacking full-text accessibility and articles containing data sourced from surveillance databases. In situations where articles had overlapping populations, preference was given to the most recent or comprehensive publication for inclusion.

*Data Extraction* For each included study, we gathered the following information: the primary author's name, publication year, study design, continent of origin, country, study duration, number of patients, number of free flaps utilized, proportion of male participants, mean age, mean time to diagnosis (following completion of radiotherapy), and the count of free flap failures.

### Quality assessment

The Quality Assessment Tools provided by the collaboration between the Universities of Newcastle, Australia, and Ottawa, Canada, were utilized by two researchers who independently conducted a meticulous evaluation of each study. The Newcastle–Ottawa Scale (NOS) and the adapted NOS for Cohort and Cross-Sectional Studies were employed, respectively. The objective of this assessment was to scrutinize each research study for potential methodological or survey implementation issues that could compromise its internal validity. The assessment involved a 'star system' in which a study was judged on three broad perspectives: the selection of the study groups, the comparability of the groups, and the ascertainment of either the exposure or outcome of interest for case–control or cohort studies (or for cross-sectional studies with the adapted tool), respectively. A study with a score ranging from 7 to 9 was considered to have low risk of bias (high quality), while a score of 4–6 indicated moderate quality, and a score of 0–3 signified high risk of bias (low quality)^12^ (Supplementary Figs. 1 and 2). The Joanna Briggs Institute (JBI) Critical Appraisal tools have been utilized for case-series. The outcomes of this appraisal are then instrumental in guiding the synthesis and interpretation of the study's results. Developed by the JBI and its collaborators, the JBI Critical Appraisal tools have been sanctioned by the JBI Scientific Committee after thorough peer review. This tool includes 10 distinct questions for the appraisal of case-series^74^.

### Statistical analysis

Model Use: The statistical analysis was carried out using the RStudio software (version: 2022.12.0 + 353) provided by RStudio Team^13^. In this analysis, the metafor package was utilized for the meta-analysis, enabling the estimation of the pooled prevalence and its corresponding 95% confidence intervals (CI) through the implementation of the DerSimonian and Laird random-effects model^14^. To calculate the prevalence, the Freeman-Tukey double arcsine transformation was employed as a part of the methodology^15^.

*Heterogeneity and Analyses* To evaluate heterogeneity across studies, a visual inspection of the forest plot and the utilization of the Cochran's Q statistic and its associated *p*-value were conducted. The magnitude of true heterogeneity in effect sizes was quantified using the Higgins I^2^ statistic and its respective 95% CI. The categorization of heterogeneity levels was as follows: 0–40% (not important), 30–60% (moderate), 50–90% (substantial), and 75–100% (considerable heterogeneity). In order to identify any influential outlying effect sizes (as initially observed in the forest plot), screening for externally studentized residuals with absolute z-values greater than two was performed, along with leave-one-out diagnostics^16^. Given the persistent moderate level of heterogeneity in this analysis, meta-regression analyses was conducted. In the performed meta-regression analysis with continuous variables, the year of publication, the proportion of males, the mean age and the mean time to diagnosis were assessed as moderators on effect sizes. However, due to limited data availability (less than ten studies for each covariate), variables such as smoking status and other factors including duration of surgery, comorbidities, alcohol consumption, obesity, and surgeon level were not included in this analysis^17^. Unless otherwise specified, a significance level of p = 0.05 (two-tailed) was considered to indicate statistical significance.

*Publication Bias* To assess the existence of publication bias, various techniques have been developed, including examinations such as Egger's test^18^, Begg's test^19^, and the analysis of funnel plots. It is worth highlighting that the tests mentioned above were originally designed to assess data from comparative studies, assuming a bias towards publishing studies with positive outcomes over those with negative results. However, in the context of a meta-analysis focusing on proportions, there is no universally agreed-upon definition of what constitutes a positive result^20^. Therefore, a qualitative evaluation was conducted to examine the potential presence of publication bias in this specific meta-analysis.

## Results and analysis

### Search results and characteristics of the included studies

**Figure 1:**
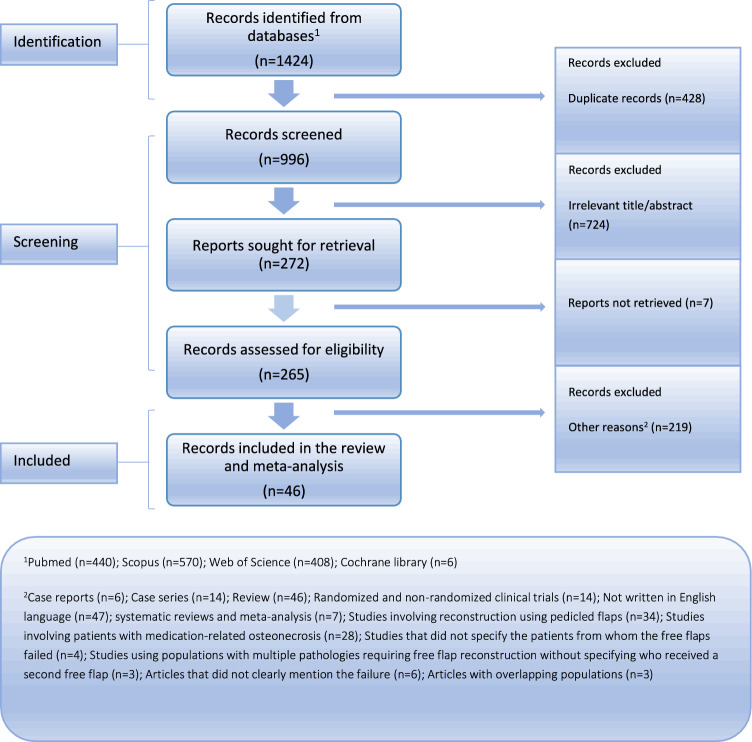
Flow chart depicting the systematic search results from the relevant studies' identification and selection.

From a total of 1424 articles, forty-six eligible studies (n = 46) involving 1292 participants and 1344 free flaps were ultimately included in this analysis. The PRISMA flowchart is depicted in Fig. 1, providing a visual representation of the systematic review and meta-analysis process.The descriptive characteristics as well as the main complications of the incorporated research are presented in Table 1. All articles were published between 1994 and 2024, with research conducted from 1982 to 2022. Five of them were identified as case series, five as cohort studies, and 36 as cross-sectional studies. The majority of the studies were conducted in North America (USA and Brazil, n = 16), followed by Europe (Belgium, Norway, Italy, Sweden, The Netherlands, France, Germany, and the UK, n = 15), Asia (Japan, Taiwan, South Korea, and China, n = 12), Australia (n = 2), and South America (Brazil, n = 1). The average percent of males was 69.1% while the mean age of participants ranged from 43 to 68.5 years (median = 54.3 years). The mean time from completion of radiotherapy to diagnosis was 39.3 months. Eight of the whole number of articles were estimated as high quality (low risk of bias)^22,24,27,31,40,42,51,54^ and the remaining ones as moderate quality (moderate risk of bias) (Supplementary Fig. 1).

**Table 1:** Main characteristics and data outcome of the included studies.

Author	Year of publication	Study design	Continent of origin	Country	Study period	Proportion of males (%)	Mean age (years)	Mean time to diagnosis (months)	Number of patients	Number of free flaps used	Number of free flaps failure
Liu^21^	2024	Cross-sectional	Asia	China	2000–2021	NA	NA	NA	193	193	32
van den Heuvel^22^	2023	Cohort	Europe	The Netherlands	1995–2021	60.7	63	35	28	28	3
Prevost^23^	2023	Cross-sectional	Europe	France	2007–2018	90	55.7	NA	10	10	3
Hurrel^24^	2023	Cross-sectional	Australia	Australia	2019–2022	NA	NA	NA	8	8	0
van Baar^25^	2021	Cross-sectional	Europe	The Netherlands	2017–2019	40	53.4	NA	5	5	0
Meleca^26^	2021	Cross-sectional	North America	USA	2008–2018	56.3	65	NA	16	16	1
Brady^27^	2021	Cohort	Europe	UK	2014–2019	60	68.5	NA	10	10	0
O'Connell^28^	2021	Cross-sectional	Europe	UK	1990–2015	75.5	58.1	NA	49	49	3
Yamashita^29^	2021	Cross-sectional	Asia	Japan	2013–2017	93.3	67	NA	15	16	1
Jenkins^30^	2021	Case-series	Europe	UK	2017–2019	NA	61	NA	12	12	2
Jin^31^	2020	Cross-sectional	Asia	China	2010–2018	NA	NA	NA	85	85	12
Danielsson^32^	2019	Cross-sectional	Europe	Sweden	2012–2015	69	62	36	16	16	0
Haffey^33^	2019	Case-series	North America	USA	2011–2014	50	66.6	NA	8	9	1
Bettoni^34^	2019	Cross-sectional	Europe	France	2003–2013	NA	57.8	57	49	55	7
Rommel^35^	2018	Cross-sectional	Europe	Germany	NA	80	59	NA	15	30	2
Lofstrand^36^	2017	Cohort	Europe	Sweden	2000–2014	66.7	58	NA	24	28	3
Akashi^37^	2017	Cross-sectional	Asia	Japan	2013–2016	90.9	66.9	NA	11	11	1
Barry^38^	2017	Cross-sectional	Europe	UK	2008–2014	NA	NA	NA	22	22	1
Chang^39^	2017	Cross-sectional	North America	USA	NA	NA	NA	NA	5	5	0
Baron^40^	2016	Cross-sectional	Europe	France	2005–2012	20	55.8	63.6	5	5	0
Kim^41^	2016	Cross-sectional	Asia	South Korea	2008–2015	62.5	60.1	NA	8	8	0
Fan^42^	2015	Cross-sectional	Asia	China	2008–2013	61.3	46.8	NA	31	31	0
Kim^43^	2015	Cross-sectional	Asia	South Korea	2009–2013	71.4	55	44	7	7	0
Shan^44^	2015	Cross-sectional	Asia	China	2003–2011	80	50.4	64.8	5	10	1
Moubayed^45^	2015	Case-series	North America	USA	1995–2013	NA	NA	NA	21	21	0
Chen^46^	2014	Cross-sectional	Asia	Taiwan	1986–2011	87	NA	NA	153	153	0
Zaghi^47^	2014	Cross-sectional	North America	USA	1995–2012	NA	NA	NA	73	73	0
Chandarana^48^	2013	Case-series	North America	USA	1999–2006	63.6	57.3	NA	11	12	0
Sawhney^49^	2012	Cross-sectional	North America	USA	1998–2010	78.4	68.5	NA	37	37	0
Hoffman^50^	2012	Cross-sectional	Australia	Australia	2005–2011	87.5	60.4	NA	8	8	0
Baumann^51^	2010	Cross-sectional	North America	USA	1998–2008	63.5	61	NA	63	75	4
Suh^52^	2010	Cross-sectional	North America	USA	1995–2009	65	NA	NA	40	40	0
Alam^53^	2009	Case-series	North America	USA	2002–2007	NA	NA	47	33	33	0
Hirsch^54^	2008	Cohort	North America	USA	1994–2004	38.1	58.1	NA	21	21	3
Curi^55^	2007	Cross-sectional	South America	Brazil	1999–2002	80	61.2	45.6	5	5	1
Chiapasco^56^	2006	Cross-sectional	Europe	Italy	1995–2002	100	51.5	NA	8	8	0
Militsakh^57^	2005	Cross-sectional	North America	USA	1998–2003	77.8	59	29	9	9	0
Gal^58^	2003	Cohort	North America	USA	1995–2002	50	63.1	NA	30	30	0
Ang^59^	2003	Cross-sectional	North America	Canada	1993–2000	66,7	62.3	NA	15	15	0
Store^60^	2002	Cross-sectional	Europe	Norway	1986–2001	52.9	49.4	NA	17	17	2
Celik^61^	2002	Cross-sectional	Asia	Taiwan	1990–2000	88.9	49.9	11.2	27	28	1
Chang^62^	2001	Cross-sectional	North America	USA	1989–1999	58.6	55.4	46	29	29	4
Shaha^63^	1998	Cross-sectional	North America	USA	NA	50	55.2	NA	6	6	0
Santamaria^64^	1998	Cross-sectional	Asia	Taiwan	NA	91.7	43	8.1	12	12	0
Nakatsuka^65^	1996	Cross-sectional	Asia	Japan	1989–1991	88.9	58	24	9	10	0
Ioannides^66^	1994	Cross-sectional	Europe	Belgium	1982–1991	71.4	56.4	NA	28	33	5

NA: not applicable.

### Prevalence of total free flap failure after free flap reconstruction for mORN

**Figure 2:**
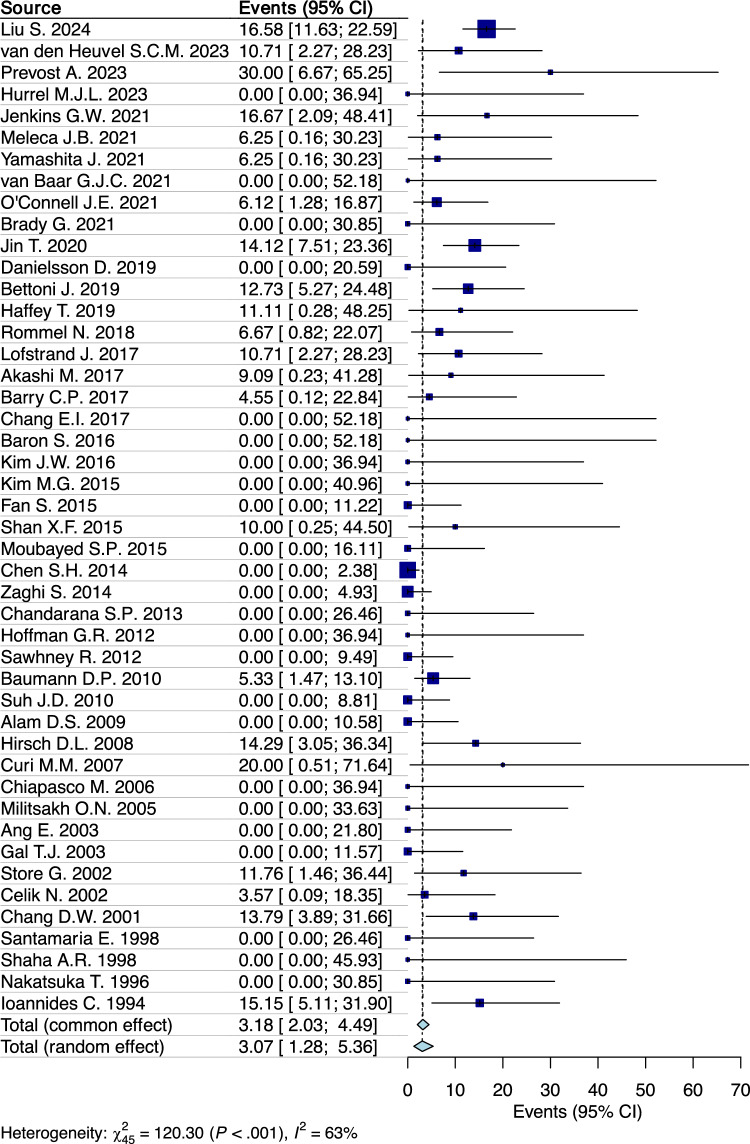
Forest plot evaluating the calculated prevalence of total free flap failure after free flap reconstruction for mORN using random-effects model.

A random-effects model analysis yielded an initial overall total free flap failure (among 1328 free flaps) prevalence of 3.1% (95% CI 1.3–5.4%) with moderate heterogeneity I^2^ = 63% (95% CI 26–65%) (p < 0.001) (Fig. 2). Further exploration of the data through influence diagnostics, alongside a detailed forest plot representation of the leave-one-out analysis outcomes, are made available in the Supplementary materials (Supplementary Figs. 2 and 3). According to these analyses, none of the studies were identified as having a significant influence on the overall results.

### Meta-regression analysis

The meta-regression analysis, which factored in continuous variables like the year of publication, the proportion of male participants, the mean age, and the mean time to diagnosis, ultimately revealed no statistically significant alterations, either positive or negative, as detailed in the Supplementary materials (refer to Supplementary Table 3).

## Discussion

Osteoradionecrosis of the jaw is defined as an exposed, nonhealing bone over a period of three months without evidence of existing malignancy. Many staging systems have been developed throughout the years in order the affected cases to be classified and treated properly. They use parameters such as the response to the therapy (HBO), clinical findings, radiological findings or combinations of them^6^. During the evolution of mORN, complications such as fractures, infection and orocutaneous fistula may occur, resulting to the patient’s declined functionality. Microvascular free flap reconstruction is the treatment of choice for severe cases. Among the free flaps used for reconstruction, free fibula flap is the most common one, while other choices include iliac crest, radial forearm flap, anterolateral thigh flap and scapular flap^67^.

Free flap failure after reconstruction for mandibular osteoradionecrosis is influenced by a myriad of factors that pose complex challenges for healthcare providers and patients. Compromised vascularity, radiation-induced tissue damage, lengthy operative times, and patient-specific factors such as malnutrition significantly affect the outcome, leading to poor tissue healing, compromised immune response, and reduced flap viability. Conditions like coagulopathies and low skeletal muscle mass further complicate recovery, underscoring the need for tailored medical strategies and highlighting their roles as predictors of surgical complications and overall survival. Additionally, gender, prior radiation therapy, poor oral hygiene, and lack of regular follow-up visits have been associated with higher rates of implant failure and complications, emphasizing the importance of comprehensive patient care. The presence of osteoradionecrosis, infection risks, and lifestyle choices such as smoking and diabetes also critically impact the success of flap reconstructions, pointing towards a complex interplay of biological, lifestyle, and treatment-related factors that determine the long-term success of mandibular reconstructions^68^.

It is crucial to identify and optimize these factors preoperatively to minimize the risk of flap failure. A thorough evaluation of the patient's overall health status, including nutritional assessment and smoking cessation counseling, should be conducted to improve outcomes. To mitigate the risk of free flap failure, a multidisciplinary approach is crucial. Preoperative evaluation and optimization of patients' medical conditions, including glycemic control and smoking cessation, are vital. Proper patient selection, based on an assessment of individual risk factors, can help identify those who are more likely to benefit from free flap reconstruction. Intraoperatively, meticulous surgical technique and anastomosis are essential. Surgeons must carefully assess the recipient vessels' quality and perform the microvascular anastomosis with precision. Monitoring of flap perfusion postoperatively through clinical examination, or microvascular imaging techniques can aid in early detection of compromised vascularity and allow for prompt intervention. Close postoperative follow-up is essential to identify any signs of flap compromise or infection. Timely management of complications, such as wound dehiscence, hematoma, or infection, is crucial to salvage the flap's viability. In cases of free flap failure, alternative reconstructive options, such as local flaps or bone grafts, should be considered to restore form and function^69–72^.

Our study provides evidence for 3.1% (95% CI 1.3–5.4%) prevalence of total free flap failure after free flap reconstruction for mORN. To the best of our knowledge, there is only a sole meta-analysis to date related to this issue in the scientific literature. Lee et al.^73^, using data from 15 studies estimate a prevalence of free flap failures (among 368 free flaps) requiring revision operations at 9.8% (95% CI 9–16%) with low heterogeneity I^2^ = 0% (p = 0.56) between studies. Our estimation based on 46 studies is lower 3.1% (95% CI 1.3–5.4%). Potential reasons for this discrepancy could be the larger number of studies used, different inclusion/exclusion criteria, quality assessment performed and the transformation of the data used in order to calculate the prevalence. Specific transformation was required since included studies in both systematic reviews (such as the study conducted from Suh et al.^52^) observed zero events of total free flap failure. More studies should be conducted in order to explore the association between the aforementioned outcomes after free flap reconstruction for mORN and potential risk factors such as high dose of ionizing irradiation, pre radiotherapy surgical treatment, primary tumor site, trauma (dental extraction), smoking status, gender, age, alcohol consumption and poor dental hygiene. In conclusion, our study provides evidence for prevalence of 3.1% (95% CI 1.3–5.4%) total free flap failure after free flap reconstruction for mORN. Our findings point to several directions for future research. Therefore, both prospective and retrospective studies need to be conducted in order this issue to be fully investigated.

### Study’s strengths and limitations

The robustness of this study lies in its rigorous methodology, which included a comprehensive exploration of the literature, meticulous selection of studies, specific criteria for inclusion and exclusion, thorough screening of eligibility, quality assessment, and systematic analysis of prevalence data from twenty-four studies. Nonetheless, several limitations were identified during the course of the study. It is crucial to acknowledge that, inherent to proportional meta-analysis, efforts were made to obtain as homogeneous a population as possible, despite the inherent challenges. Consequently, several assumptions had to be made. The analysis included studies even when they did not explicitly mention the definition of ORN used to identify cases. In such instances, a thorough examination of the entire manuscript was undertaken to ascertain its suitability. Moreover, this meta-analysis amalgamated results from studies employing varying reconstruction techniques, and encompassed a broad spectrum of ages, genders, and comorbidities. However, studies focused on assessing prevalence in specific populations, such as those exclusively comprising individuals with diabetes, were intentionally excluded. Additionally, only studies that explicitly defined and identified cases of free flap failure (designating as failures those instances where salvage efforts failed, leading to non-viable tissue (flap necrosis)) were considered. Notably, there was a moderate level of unexplained heterogeneity observed in relation to the prevalence of total free flap failure. The diversity of outcomes across the studies included in this meta-analysis can largely be attributed to the inherent nature of such studies. Additionally, various conceivable risk factors, including but not limited to diabetes, impaired immune response, prolonged operative duration, obesity, patient age, gender, additional surgical interventions, as well as tobacco and alcohol consumption habits, may introduce bias in the prevalence of free flap failure following mandibular reconstruction. Furthermore, the analysis faced limitations due to a lack of detailed information, making it impossible to categorize complications by free flap type. Due to the scant number of studies addressing each of these factors (fewer than ten for each) they were excluded from the meta-regression analysis. Additionally, the study's inclusion criteria were restricted to observational studies published in English, potentially skewing the evidence base and excluding comprehensive representation of studies conducted in non-English speaking countries or in locales with limited resources.

### Supplementary Information

Supplementary Information.

## Data Availability

Literature and Rstudio data are available from the corresponding author on reasonable request.
